# Chemical Characterization of *Sambucus nigra* L. Flowers Aqueous Extract and Its Biological Implications

**DOI:** 10.3390/biom11081222

**Published:** 2021-08-17

**Authors:** Pedro Ferreira-Santos, Helder Badim, Ângelo C. Salvador, Armando J. D. Silvestre, Sónia A. O. Santos, Sílvia M. Rocha, Ana M. Sousa, Maria Olívia Pereira, Cristina Pereira Wilson, Cristina M. R. Rocha, José António Teixeira, Cláudia M. Botelho

**Affiliations:** 1 CEB—Centre of Biological Engineering, Campus de Gualtar, University of Minho, 4710-057 Braga, Portugal; helderbadim@hotmail.com (H.B.); anamargaridasousa@deb.uminho.pt (A.M.S.); mopereira@deb.uminho.pt (M.O.P.); cpereira@bio.uminho.pt (C.P.W.); cmrocha@ceb.uminho.pt (C.M.R.R.); jateixeira@deb.uminho.pt (J.A.T.); 2 CICECO—Aveiro Institute of Materials, Chemistry Department, Campus de Santiago, University of Aveiro, 3810-1930 Aveiro, Portugal; angelomcsalvador@gmail.com (Â.C.S.); armsil@ua.pt (A.J.D.S.); santos.sonia@ua.pt (S.A.O.S.); 3 Departamento de Química & LAQV-REQUIMTE, Universidade de Aveiro, 3810-193 Aveiro, Portugal; smrocha@ua.pt; 4 Department of Biology, University of Minho, Campus de Gualtar, 4710-057 Braga, Portugal

**Keywords:** elderflower, GC-MS, HPLC-MS, chemical profile, phenolic compounds, cytotoxicity, antibacterial activity, antioxidant activity

## Abstract

The main goal of this study was to chemically characterize an aqueous *S. nigra* flower extract and validate it as a bioactive agent. The elderflower aqueous extraction was performed at different temperatures (50, 70 and 90 °C). The extract obtained at 90 °C exhibited the highest phenolic content and antiradical activity. Therefore, this extract was analyzed by GC-MS and HPLC-MS, which allowed the identification of 46 compounds, being quercetin and chlorogenic acid derivatives representative of 86% of the total of phenolic compounds identified in hydrophilic fraction of the aqueous extract. Naringenin (27.2%) was the major compound present in the lipophilic fraction. The antiproliferative effects of the *S. nigra* extract were evaluated using the colon cancer cell lines RKO, HCT-116, Caco-2 and the extract’s antigenotoxic potential was evaluated by the Comet assay in RKO cells. The RKO cells were the most susceptible to *S. nigra* flower extract (IC_50_ = 1250 µg mL^−1^). Moreover, the extract showed antimicrobial activity against Gram-positive bacteria, particularly *Staphylococcus aureus* and *S. epidermidis*. These results show that *S. nigra*-based extracts can be an important dietary source of bioactive phenolic compounds that contribute to health-span improving life quality, demonstrating their potential as nutraceutical, functional foods and/or cosmetic components for therapeutic purposes.

## 1. Introduction

Natural products are known to provide an endless source of bioactive compounds that can be used in medicine. Plant products have been used with variable success to prevent and delay the onset and progression of aging-associated diseases, increasing health-span and improving life quality [1]. Recently, there has been an interest in so-called nutraceuticals and functional foods. The functionalization of foods and beverages with plant-based extracts, strongly affected the natural supplement market. This market registered an exponential growth in the production and commercialization of food supplements, especially those which are plant-based [2]. There is evidence that a diet rich in plant derivatives is usually associated with a lower risk of diseases, such as neurological disorders, diabetes, cancer, cardiovascular and inflammatory diseases [3,4,5]. This may be due to the numerous bioactive substances, both of low molecular weight, like phenolic compounds, and high molecular weight, as polysaccharides [6].

Dietary polyphenols are one of the most important classes of natural antioxidants and chemopreventive agents found in the human diet. Currently, there is evidence that a dietary rich in phenolic compounds can improve human health by lowering risk and preventing the onset of cancer, metabolic disorders, cardiovascular and degenerative diseases [6]. The ability of dietary polyphenols to restore redox homeostasis and prevent inflammation by enhancing the activity of endogenous antioxidant enzymes has been reported in the literature [7,8]. In recent years, these compounds are being thoroughly studied due to their anti-cancer activity and low toxicity [9], as well as their role as a multi-target agents in the prevention and treatment of cancer.

The current agro-industrial market pursues the desire of the consumer by developing high value-added products that are particularly effective in disease prevention, as well as in promoting healthy aging [1,10]. In the last decades, *Sambucus nigra* L. flowers (elderflower) has received increased attention due to the multitude of flowers on the plant tree and their rich chemical composition with a large number of bioactive ingredients, including proteins, vitamins, minerals, terpenes, sterols and polyphenols [11,12]. These compounds may contribute to nutritional and pharmacological actions, indicating a set of potential health benefits related with the *S. nigra* (bio)products consumption [13]. What is surprising is the fact that, although *S. nigra* flowers products are very popular in traditional medicine (e.g., treatment of flu and cold, respiratory infections, etc.) and daily use, there are few commercial products on the market based on *S.nigra.* The identification of molecules present in the extract can provide insights on its potential for disease prevention or treatment. These properties are often related to polyphenols, especially phenolic acids, flavonols and proanthocyanidins, which are considered key players regarding antioxidant, anti-cancer and antimicrobial activities [13,14].

Thus, the main goal of this study is to chemically characterize *S. nigra* flower aqueous extracts by different techniques (like spectrophotometry, liquid and gas chromatography), as well as to assess its biological potential (antioxidant, antimicrobial and anti-proliferative/anti-genotoxic).

## 2. Materials and Methods

### 2.1. Chemicals

Folin-Ciocalteu reagent, 6-hydroxy-2,5,7,8-tetramethylchroman-2-carboxylic acid (Trolox), sodium carbonate (Na_2_CO_3_), 2,2-diphenyl-1-picrylhydrazyl (DPPH), caffeic acid, luteolin, hydrogen peroxide (H_2_O_2_), Thiazolyl Blue Tetrazolium Bromide (MTT), Trypic Soy Broth, Sabouraud Dextrose Broth, Mueller Hinton Broth and RPMI. Eagle’s Minimum Essential Medium (MEM), fetal bovine serum (FBS), RPMI-1640 medium, Dulbecco’s modified Eagle’s medium (DMEM), Non-essential Amino Acids (NEAA), and all HPLC and GC standard markers were obtained from Sigma-Aldrich (St. Louis, MO, USA). All other chemicals used were of analytical grade and water was ultra-pure.

### 2.2. Extraction Conditions and Extracts Preparation

The flower of *S. nigra* was purchased dry (humidity less than 10%) in a local supermarket (Braga, Portugal). Extraction process was performed using 5 g of dry flower with 150 mL distilled water (33.33 g L^−1^) using 250 mL cylindrical reactors duly protected from light. A thermostatic bath at controlled temperature and agitation of 150 rpm for 30 min was used. Extraction temperatures of 50 °C, 70 °C and 90 °C were tested. After extraction, the solid was separated from the liquid extract, and the extract was centrifuged at 9000 rpm, 5 °C for 10 min and filtered with 0.22 µm filter paper. Liquid extracts resulting from each extraction were used to determine the total phenolic content (TPC) and antioxidant activity. The remaining samples were freeze-dried and stored at 4 °C in the dark until further use. This experiment was performed in triplicate.

### 2.3. Chemical Characterization of Extracts

#### 2.3.1. Total Phenolic Content (TPC)

Total phenolic content was determined according to the Folin-Ciocalteu method using caffeic acid as standard. The reaction mixture was composed by 5 µL of liquid extract (without freeze-dried) resulting from each extraction, with 60 µL of sodium carbonate 15% (*w*/*v*) and 20 µL Folin-Ciocalteu reagent. Finally, 200 µL distilled water was added to the mixture and incubated at 60 °C for 10 min. The absorbance was measured at 700 nm by an UV/vis spectrophotometer (Synergy HT, BioTek Instruments, Inc., Winooski, VT, USA). Caffeic acid was used to perform the standard curve (1000–10 mg L^−1^, *R*^2^ = 0.998) and total phenolic content was expressed as milligrams of caffeic acid equivalents per gram of dry *S. nigra* flower (mg CAE g^−1^). Analyses were performed in triplicate.

#### 2.3.2. DPPH Scavenging Activity

Antioxidant activity of *S. nigra* flower aqueous extract was determined by 2,2-diphenyl-1-picrylhydrazyl (DPPH) radical scavenging assay. The DPPH scavenging assay is frequently used to assess the free radical scavenging activity of antioxidant compounds, being acknowledged as one of the easiest colorimetric assays to evaluate the antioxidant potential [15]. Trolox, prepared in methanol, was used as standard (0.25–0.015 mmol, *R*^2^ = 0.995). The mixture composed by 20 µL of liquid extracts (without freeze-dried) resulting from each extraction and 200 µL DPPH solution (150 µM, dissolved in 80% methanol) was incubated in the dark at room temperature for 60 min. The absorbance of the mixture was measured at 515 nm by an UV/vis spectrophotometer. Antioxidant activity or the ability of the extract to scavenge DPPH free radical was expressed as millimoles of Trolox equivalent per gram of dry *S. nigra* flower (mmol TE g^−1^). Analyses were performed in triplicate.

#### 2.3.3. GC-MS Analysis of Lipophilic Fraction

The lipophilic fraction of *S. nigra* aqueous extract was recovered and analyzed. For this, 30 mL of *S. nigra* liquid extract (from aqueous extraction at 90 °C) was mixed with 90 mL dichloromethane for 30 min, and the two phases were separated. The organic phase was recovered, the solvent was evaporated to dryness in a rotatory evaporator and the solid extract weighted. The lipophilic fraction obtained was used and converted into trimethylsilyl (TMS) derivatives according to methodology of Domingues and coworkers [16]. GC–MS analyses were performed using a GC-MS QP2010 Ultra equipped with a Thermo Scientific DSQII mass spectrometer (Shimadzu Scientific Instruments, Inc., Columbia, MD, USA) using helium as carrier gas (35 cm s^−1^) equipped with a DB-1 MS capillary column (30 m × 0.32 mm × 0.25 µm). The chromatographic conditions were as follows: initial temperature 80 °C for 5 min, temperature rate of 4 °C min^−1^ up to 260 °C, and 2 °C min^−1^ until the final temperature 285 °C, then maintained at 285 °C for 13 min, injector temperature of 250 °C; transfer-line temperature 290 °C, split ratio: 1:50. The MS was operated in the electron impact mode with electron impact energy of 70 eV and data collected at a rate of 1 scan s^−1^ over a range of *m*/*z* 33–700. The ion source was maintained at 250 °C. Compounds were identified as TMS derivatives by comparing their mass spectra with the GC–MS spectral library (Wiley-NIST Mass Spectral Library 1999), with literature MS fragmentation [17,18,19]. For quantitative analysis, tetracosane (C_24_H_50_) was used and the response factor of pure standards representative of each family were determined, namely ferulic acid to evaluate phenolic acids, palmitic acid for fatty acids and nonadecan-1-ol for alcohols [19]. The respective response factors needed to obtain correct quantification of the peak areas were calculated based on three standards concentrations as an average of three GC–MS runs of each concentration using GCMS solution Software (Shimadzu, Columbia, MD, USA). Three independent aliquots were derivatized and submitted to GC–MS analysis. Each aliquot was injected in duplicate. The presented results are the average of the concordant values obtained for each sample (*n* = 3). Compound content was expressed as micrograms of respective compound per gram of dry *S. nigra* flower or extract (µg g^−1^).

#### 2.3.4. HPLC-MS Analysis of Phenolic Components

To carry out the qualitative analysis of *S. nigra* aqueous extract a solution of 10 mg mL^−1^ of lyophilized extract was prepared, using water as solvent, and subsequently filtered with a 0.22 μm PTFE syringe filter. Individual phenolic compounds were analyzed on a Thermo Finnigan Surveyor HPLC (Thermo Fisher Scientific Inc., Waltham, MA, USA) system with a diode array detector (DAD). Spectra of the compounds were recorded between 200 and 600 nm, identification and quantification of flavanols, hydroxycinnamic acid derivatives and flavanone were made at 320 nm and 360 nm for flavonols. The used column was a Gemini C18 (150 × 4.6 mm × 3 μm, from Phenomenex^®^, Alcobendas, Spain), operated at 25 °C. The elution solvents were 0.1% formic acid in acetonitrile (ACN) (A), 1% ACN and 0.1% of formic acid in distilled water (B), and ACN (C). Samples were eluted according to a linear gradient from 5% to 20% B in the first 15 min, followed by a linear gradient from 20% to 30% B for 5 min, then an isocratic mixture for 5 min, followed by a linear gradient from 30% to 90% B for 5 min and then an isocratic mixture for 15 min before returning to the initial conditions. The injection amount was 20 μL and flow rate 0.6 mL min^−1^. All phenolic compounds were identified using a mass spectrometer with electrospray ionization (ESI) operating in negative ion mode. The analyses were carried out using full scan data-dependent MSn scanning from *m*/*z* 115 to 1500. The injection volume was 1 μL and the flow rate maintained at 0.6 mL min^−1^. The capillary temperature was 250 °C, the sheath gas and auxiliary gas were 60 and 15 units, respectively; the source voltage was 3 kV and normalized collision energy was between 20−35%. Spectral data were elaborated using the Excalibur software.

Contents of phenolic compounds and flavonols were calculated from peak areas of the sample at maximum wavelength absorption and using similar compounds as standards (chlorogenic acid and rutin, respectively), expressed in µg g^−1^ of *S. nigra* flower or extract. The presented results are the average of the concordant values obtained for each sample (*n* = 3).

Calibration curves between 0 to 50 µg mL^−1^ of chlorogenic acid (*R*^2^ = 0.997) and rutin (*R*^2^ = 0.996) were used for quantification. Limits of detection (LOD) and quantification (LOQ) were also estimated showing values of 3.2 and 10.6 µg mL^−1^ of chlorogenic acid, respectively, and 3.3 and 10.9 µg mL^−1^ of rutin, respectively.

### 2.4. Biological Evaluation

#### 2.4.1. Antiproliferative Activity in Colon Cancer Cell Lines

Several cell lines were used to assess the biological potential of the aqueous extract: RKO (human colon carcinoma, ATCC^®^ CRL-2577™), HCT116 (human colorectal carcinoma, ATCC^®^ CCL-247™), Caco-2 cells (human colorectal carcinoma, ATCC^®^ HTB-37™).

The RKO cell line was cultured in ATCC Eagle’s Minimum Essential Medium (MEM) in the presence of 10% (*v*/*v*) fetal bovine serum (FBS) and 1% penicillin/streptomycin solution at 37 °C in 5% CO_2_. HCT116 cells were cultured in RPMI-1640 medium supplemented with 10% (*v*/*v*) FBS and 1% (*v*/*v*) penicillin/streptomycin solution at 37 °C in 5% CO_2_. Caco-2 cells were cultured in Dulbecco’s modified Eagle’s medium (DMEM) supplemented with 10% (*v*/*v*) FBS, 1% (*v*/*v*) Non-essential Amino Acids (NEAA), under a humidified atmosphere containing 5% CO_2_ at 37 °C.

Cells were plated in 48-well plates at a density of 5 × 10^4^ cells mL^−1^ for RKO and HCT116 cell lines and 3 × 10^4^ cells mL^−1^ for Caco-2 cell line and left to adhere overnight. The previously lyophilized extract (obtained at 90 °C) was dissolved in culture medium to obtain concentrations ranging from 0–2500 µg mL^−1^ immediately before use in cell culture, and the cells incubated with the extract for 48 h at 37 °C and 5% CO_2_. Control cells were grown with complete medium. The cellular viability was assessed using the Thiazolyl Blue Tetrazolium Bromide (MTT) reduction assay according to Simões et al. [20]. MTT produces a yellowish solution that is converted to dark blue, water-insoluble MTT formazan by mitochondrial dehydrogenases of living cells. The blue crystals are solubilized with acidified isopropanol and its intensity is measured in an UV/vis spectrophotometer at a wavelength of 570 nm. Each experiment was performed in triplicate.

The results were expressed as a percentage of controls (cells grown in complete medium, taken as 100% viability) and the respective IC_50_ values were calculated using GraphPad Prism 7 using the curve fit—Dose-response—Stimulation.

#### 2.4.2. Antigenotoxic Effects Assessed by the Comet Assay

The alkaline version of the comet assay (single cells gel electrophoresis) [21] was used to assess DNA strand breaks in RKO cells and both DNA damage and repair capacity were evaluated as described Ramos et al. [22].

Briefly, after treatment, RKO cells were washed with PBS and trypsinized. Cells were resuspended, and 10 μL of cell suspension was mixed with 40 μL low melting point agarose. About 5000 cells mL^−1^ were placed on a slide pre-coated with normal melting point agarose. Slides were placed on ice for 10 min followed by exposure to a lysis solution (2.5 M NaCl, 100 mM Na_2_EDTA, 10 mM Tris, pH 10) plus 1% Triton X-100 for 1 h at 4 °C. After lysis, slides were placed in an electrophoresis chamber with electrophoresis solution (300 mM NaOH, 1 mM Na_2_EDTA, pH 13) for 40 min at 4 °C for the DNA to unwind before electrophoresis which was run for 20 min at 25 V and 300 mA. Later, for neutralization, washing and fixation, slides were removed from electrophoresis chamber and washed with a neutralizing buffer (PBS) for 5 min and washed two times in distillated water. Finally, fixation was performed by placing slides for 10 min in 70% ethanol and 10 min in absolute ethanol, and slides left to dry at room temperature. For the analysis of comet images, slides were stained with SYBR Gold solution for 30 min at 4 °C, followed by analysis in a fluorescence microscope. Visual score analysis was used to calculate de parameter% tail intensity.

In order to evaluate the effect of exposure to *S. nigra* aqueous extract and luteolin on prevention of DNA oxidative damage, RKO cells were incubated with complete medium supplemented with 200 and 400 µg mL^−1^ of extract (or luteolin, 20 µM) for 24 or 48 h at 37 °C and 5% CO_2_ and then washed with PBS and exposed to H_2_O_2_ (75 μM) for 5 min on ice to induce DNA and submitted to the comet assay.

For the DNA Cellular Repair Assay, cells were treated as previously and then the H_2_O_2_ was removed by washing cells with PBS and cells were allowed to recover in fresh culture medium for 5 min at 37 °C before the comet assay. The remaining DNA damage was then evaluated by the comet assay, as previously described. The percentage of DNA repair was calculated by the following equation:

% of repair DNA damage=(T0−TR)/T0×100

*T*0—% DNA damage before recovery period

*TR*—% DNA damage after 5 min of recovery time

#### 2.4.3. Antimicrobial Activity

The antimicrobial activity of the *S. nigra* flower aqueous extract was established by determining the minimum bactericidal concentration (MBC) and the minimum fungicidal concentration (MFC) using the microdilution method following the recommendations of the Clinical and Laboratory Standards Institute [23]. MBC was tested against gram-negative bacteria, including *Pseudomonas aeruginosa* (PAO1), *Klebsiella oxytoca* (ATCC 13182) and *Klebsiella pneumoniae* (ATCC 11296) and against gram-positive bacteria, including *Staphylococcus aureus* (ATCC 25293) and *Staphylococcus epidermidis* (ATCC 12228). MFC was also carried out against *Candida albicans* (SC 5314). Bacterial and fungi cells were grown on Trypic Soy Broth and Sabouraud Dextrose Broth, respectively, overnight at 37 °C, 120 rpm.

MBC and MFC were assayed using a 96-well plate with different concentrations of plant extract ranging from 64 to 33,000 µg L^−1^ prepared in Mueller Hinton Broth or RPMI, for bacteria or fungi, respectively. The bacteria and fungi were added to the wells to obtain a final concentration of 5 × 10^5^ CFU mL^−1^ and 1.5 × 10^5^ cell mL^−1^, respectively. Microbial suspensions were incubated at 37 °C, 120 rpm for 18–21 h. Afterward, cultures were plated onto Mueller Hinton Agar (for bacteria) and Sabouraud Dextrose Agar (for fungi). MBC and MFC were determined by the minimal concentration of extract required to eradicate the bacteria and fungi, respectively. All tests were performed in triplicate.

### 2.5. Statistical Analysis

The data analysis was performed using GraphPad Prism 7 software (GraphPad Software Inc., San Diego, CA, USA). Results are represented as mean ± standard deviation (SD), and statistical comparisons were calculated with a 95% confidence interval. Parametric tests were applied since all data sets presented Gaussian distributions. One-way analysis of variance (ANOVA) was used for comparison of more than two means. Two-way ANOVA was used to examine the influence of two independent variables on one continuous variable. Of each test results a *p*-value indicates the significance value of each tested sample. This significance is indicated in the figures with *p* < 0.05 (*), *p* < 0.01 (**), *p* < 0.001 (***) or *p* < 0.0001 (****).

## 3. Results

### 3.1. Total Phenolic Content and Antioxidant activity

The aqueous extraction at 90 °C for 30 min showed an extraction yield of 30.14% (g 100 g^−1^ of flower dry weight), higher than the product resulting from extractions at 50 °C and 70 °C, which were 20.32% and 25.09%, respectively.

**Figure 1:**
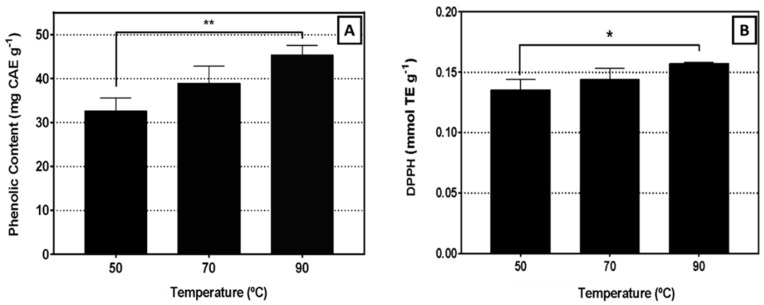
Phenolic content (**A**) and radical scavenging activity (DPPH) (**B**) of *Sambucus nigra* L. flower aqueous extracts at different extraction temperatures. Values are the mean of three independent experiments ± SD. * *p* < 0.05, ** *p* < 0.01.

The chemical composition and antioxidant potential are crucial parameters for establishing the health benefits of food products. The TPC and antioxidant capacity of the three obtained aqueous extracts were evaluated. As can be seen in Figure 1A,B, the extracts produced at higher temperatures have a higher TPC, as well as a greater radical scavenging activity evaluated by DPPH assay. Therefore, all the subsequent analyses (chemical characterization and its biological implications) were performed on the aqueous extract prepared at the highest temperature (90 °C) based on the highest extraction yield of antioxidant phenolic compounds.

### 3.2. Chemical Characterization of S. nigra Flower Extract

Several bioactive compounds, or phytochemicals, with different characteristics comprise plant extracts, a fact which poses a huge challenge for their characterization and separation. A detailed characterization of the aqueous extract obtained at 90 °C was performed using GC-MS and HPLC-MS, which allowed for a qualitative and quantitative analysis. In this case, it was possible to analyze the extract in the volatile, medium-volatile, low-volatile and non-volatile substances present in the *S. nigra* flower extract.

#### 3.2.1. Chemical Composition of the Lipophilic Fraction by GC-MS

In order to determine the lipophilic composition *S. nigra* aqueous extract, a sample was submitted to liquid-liquid extraction with dichloromethane, a solvent that is fairly selective for the isolation of the lipophilic components from plant materials and analyzed by GC-MS after conversion into trimethylsilyl derivatives [24].

The lipophilic fraction yield of *S. nigra* aqueous extract was 0.42% (g 100 g^−1^ of flower dry weight) representing 1.39% of the total extract. The identification and quantification data is summarized in Table 1.

The GC-MS analysis revealed the presence of 31 compounds distributed over seven chemical families. Flavonoids accounted for 34.58% of all identified compounds, followed by phenolic acids, which account for 19.44% of the identified compounds, fatty acids (C_6_–C_24_) (14.30%), sugars (11.80%), monoterpenes (7.8%), glicerol (6.53%) and other minor compounds like as aminoacid derivative (methyl leucinate) and 2–3-butanediol.

The only flavonoid identified in the lipophilic fraction was naringenin, accounting for a total of 389.14 µg g^−1^ of dry flower aqueous extract. Other phenolic compounds quantified in the lipophilic fraction of extract were benzoic acid, ρ-anisic acid, cinnamic acid, 4-coumaric acid, ferulic acid and tyrosol.

The fatty acids present were mainly medium-chain fatty acids (MCFAs) like hexanoic (9.94 µg g^−1^ of total extract) and nonanoic (15.48 µg g^−1^) acids, and long-chain fatty acids (LCFAs) like palmitic (59.08 µg g^−1^), oleic (18.95 µg g^−1^), stearic (38.25 µg g^−1^), behenic (17.61 µg g^−1^) and a residual amount of lignoceric acid (1.66 µg g^−1^). The palmitic acid was the most abundant fatty acids component, representing 40.62% of the total identified fatty acids.

#### 3.2.2. Phenolic Composition of the Aqueous Extract by HPLC-MS

The phenolic composition of the *S. nigra* flower aqueous extract was studied by high-pressure liquid chromatography-tandem mass spectrometry (HPLC-MS^n^).

Phenolic compounds were identified by comparing retention times with standards and by using tandem mass spectrometric detection and quantified by HPLC-UV using calibration curves of reference compounds representative of each chemical family. The phenolic compounds identified, as well as their retention time, the maximum UV wavelengths absorption, the corresponding [M–H]^−^ ions and the key MS^n^ fragmentation production ions relevant for their identification are summarized in Table 2.

**Figure 2:**
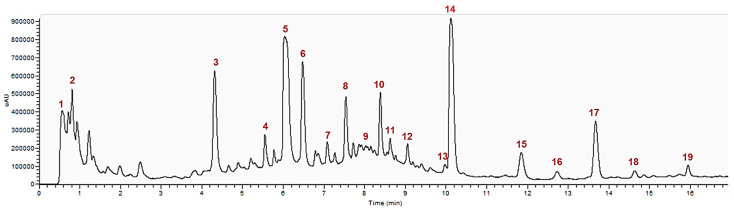
HPLC-PDA profile chromatogram of aqueous extract of *S. nigra* flowers. Time of retention is expressed in minutes. Compounds: **1**—Quinic Acid; **2**—p-Coumaroyl-caffeoylquinic Acid; **3**—Caffeoylquinic Acid Isomer; **4**—Dicaffeoylquinic Acid; **5**—Caffeoylquinic Acid Isomer; **6**—Caffeoylquinic Acid Isomer; **7**—Quercetin dihexoside Isomer; **8**—Coumaroylquinic Acid; **9**—Coumaroylquinic Acid derivative; **10**—Feruloylquinic Acid; **11**—Quercetin dihexoside Isomer; **12**—Quercetin trisaccharide; **13**—Isorhamnetin derivative; **14**—Quercetin-3-rutinoside; **15**—Quercetin-acetyl glucoside; **16**—Kaempferol rutinoside; **17**—Isorhamnetin-rutinoside; **18**—Kaempferol residue; **19**—Isorhamnetin acetylhexoside.

**Figure 3:**
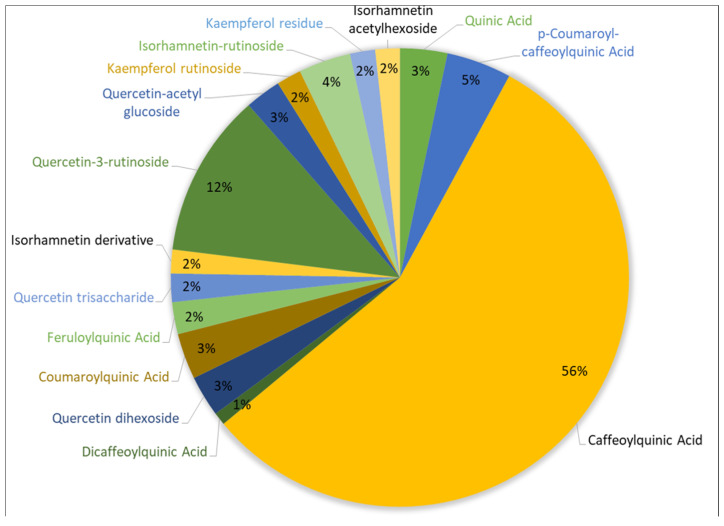
Pie chart of phenolic compounds identified in *S. nigra* flower aqueous extract (related to Table 2).

Nineteen phenolic compounds were identified (Figure 2), representing a total of 16.32 mg g^−1^ of *S. nigra* dry flower (52 mg/g dry extract). Caffeoylquinic acid is the major phenolic acid and quercetin the major flavonol found in *S. nigra* aqueous extract, accounting for 9146.5 (±362.6) and 1887.7 (±269.1) µg g^−1^ of *S. nigra* flower, respectively. Other (derivatives) compounds such as kaempferol, isorhamnetin, ferrulic acid, caffeic acid and coumaric acid are also present in the aqueous extract of *S. nigra* (see Table 2 and Figure 3).

### 3.3. Biological Activities of S. nigra Flower Extract

#### 3.3.1. Antiproliferative Activity

The antiproliferative activity of aqueous extract of *S. nigra* flowers was evaluated in three human colorectal cancer cell lines (Caco-2, RKO and HCT-116). The results are expressed as IC_50_ (µg mL^−1^), defined as the concentration that inhibits the cell growth by 50% (Table 3).

*S. nigra* flower extract exhibited a dose-response relationship in all cell lines tested and the most pronounced effect of flower extract was observed in RKO, which had the lowest IC_50_ value (1250 ± 60 µg mL^−1^), while the less susceptible cell line to *S. nigra* extract cytotoxicity were HCT-116 cells (3441 ± 230 µg mL^−1^) (Supplementary material Figure S1).

Taking into account the obtained results, the RKO cell line, the most susceptible to *S. nigra* flower extract, was selected for further tests of antigenotoxic and antimicrobial effects.

#### 3.3.2. Antigenotoxic Effects Assessed by the Comet Assay

**Figure 4:**
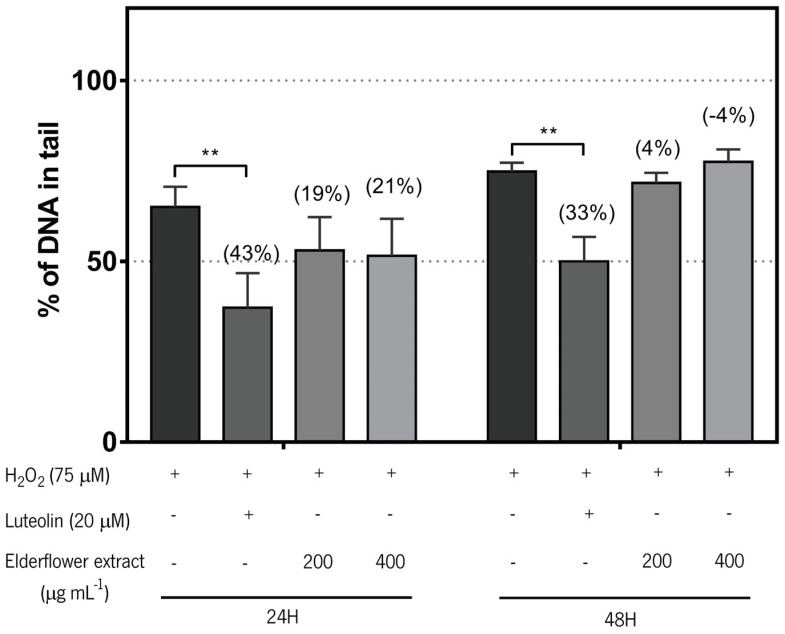
Effects of 24 and 48 h of pre-treatment with luteolin-7-glucoside (Luteolin, 20 µM) or *S. nigra* (elderflower) extract (200 and 400 µg mL^−1^) on oxidative DNA damage induced by 75 µM H_2_O_2_ (5 min, on ice) in RKO cells. [+]—compound present; [-]—compound absent; ()—percentage of protection regarding to the respective control. Results are expressed as mean ± SD, of at least three independent experiments. ** *p* < 0.01.

With regard to the effects of the extract on oxidative DNA damage protection, the comet assay was used after 24 or 48 h of pre-treatment with *S. nigra* flower extract followed by 5 min exposure to the H_2_O_2_. The results presented in Figure 4 show that exposure to H_2_O_2_ induces DNA damage (more than 50% DNA in comet tail) in RKO cells and that pre-treatment with luteolin-7-glucoside (Luteolin), used as positive control, results in a significantly lower percentage of DNA in comet tail, indicating that the compound protected the cell’s DNA from the damaging agent H_2_O_2_. The effects of pre-treatment with the flower extract were not significantly different from the control. It is noteworthy that at 24 h of pre-treatment there is a decrease in the DNA damage of approximately 20%, which may indicate some protective effect.

**Figure 5:**
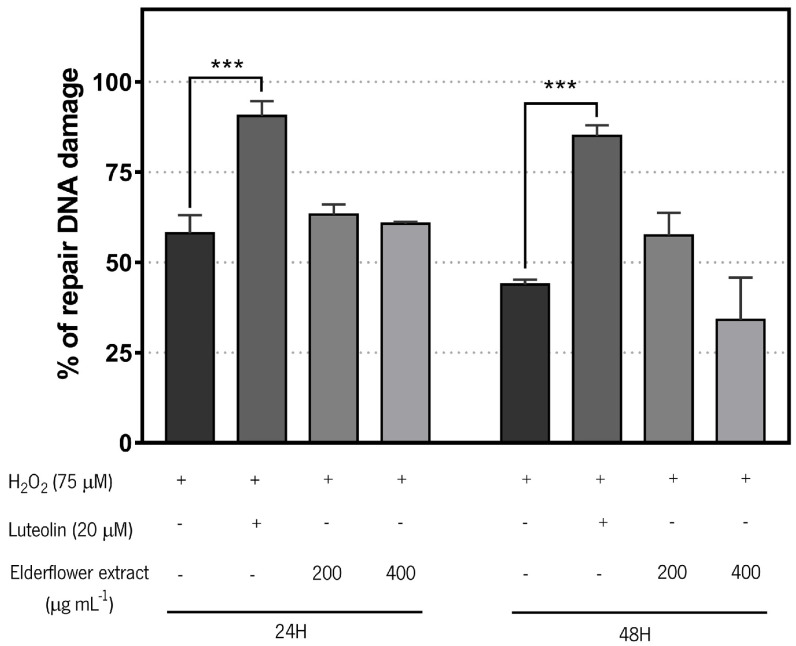
Extent of repair of 75 µM H_2_O_2_-induced damage in RKO cells after pre-incubation during 24 and 48 h with *S. nigra* (elderflower) aqueous extract (200 and 400 µg mL^−1^) or luteolin (20 µM) followed by 5 min of recovering time. [+]—compound present; [-]—compound absent. Results are expressed as mean ± SD, of at least three independent experiments. *** *p* < 0.001.

When considering the effects of the extract on induction of DNA repair capacity, the cells are pre-treated with the extract (or Luteolin), exposed for 5 min to H_2_O_2_, and allowed to recover in fresh medium for 5 min. The rate in which cells repair their DNA reflects the effect of the previous treatment. The values were calculated relative to the control cells (not pre-treated) and the higher the bar in the graph (Figure 5), the faster the repair is occurring. As can be seen in Figure 5, only the pre-treatment with luteolin (positive control) results in significant induction of repair capacity.

#### 3.3.3. Antimicrobial Activity

The antimicrobial activity of *S. Nigra* flower aqueous extract was evaluated based on MBC and MFC against several pathogen organisms. As presented in Table 4, gram-negative bacteria (*P. aeruginosa*, *K. pneumoniae*, *K. oxytoca*) and fungi exhibited values of MBC and MFC, respectively, higher than 33,000 µg mL^−1^. However lower concentrations of extract were needed to eradicate gram-positive bacteria (*S. aureus* and *S. epidermidis*), displaying MBC values between 8300 to 4100 µg mL^−1^. Therefore, gram-positive bacteria seemed to be susceptible to *S. nigra* aqueous extract.

## 4. Discussion and Conclusions

### 4.1. Phenolic Content and Antioxidant Activity

Natural products such as plant-based extracts provide numerous opportunities for new drug discoveries because of an unmatched availability of chemical diversity. In this sense, *S. nigra* has been subjected of enormous interest, and several studies involving this plant showed huge benefits of leaves, flower and fruit to human health [25]. However, in order to obtain the desired components, the extraction procedure is a crucial step. Based on the purpose of this work, to produce a natural extract from *S. nigra* flowers, water was considered the most suitable solvent, not only due to its non-toxicity, but also because it is described as one of the best solvents to produce *S. nigra* extracts with high free radical scavenging activity [26]. However, there are several factors that might affect extraction process, such as matrix properties (plant part), temperature, pH, pressure and time [27]. In this work, the influence of temperature (50, 70 and 90 °C) on the extraction process was evaluated by considering both TPC and antioxidant potential, as it can be seen in Figure 1A,B (TPC and antioxidant potential, respectively). The results demonstrated that high temperatures, particularly 90 °C, have the highest phenolic content (*p* < 0.01) and antiradical activity (*p* < 0.05), compared to extracts obtained at 50 °C. These two parameters may be correlated, as phenolic compounds are molecules with good scavenging activity and metal chelators, due to the hydroxyl groups in their structure [28,29,30]. This implication was also confirmed by Gonçalves and co-authors [31], who found a high relationship between the antioxidant activity (DPPH assay) and TPC of infusions prepared with Mediterranean medicinal plants. However, it is important to note that the TPC estimated through this method may not correspond to an absolute measure of phenolic content, but demonstrates their reducing capability relative to an equivalent reducing capacity of caffeic acid. Nevertheless, the high content of TPC quantified by the Folin-Ciocalteu method was also corroborated by the great phenolic content found in the chemical chromatographic characterization of this extract (Table 2). Moreover, these results are in accordance with some studies already reported, indicating that high temperatures tended to improve the recovery of phenolic compounds and increase their content in the final extract [32]. This may occur through thermal destruction of cell walls and subcellular compartments during heating, favoring the release of these compounds [33]. Viapiana and coworkers [34] reported that the TPC of infusions from 13 *S. nigra* flowers ranged from 15.2 to 35.6 mg CAE g^−1^ flower (and flavonoids content from 5.27 to 13.19 mg rutin equivalent g^−1^), values which are slightly lower than those obtained in this work, possibly due to the extraction conditions (room temperature for 15 min).

Antioxidants are known to be beneficial for human health by diminishing the oxidative stress. Furthermore, the bioactive properties (as antioxidant) of the isolated bioproducts may differ due to a number of factors, such as the conditions of handling, storage of postharvest of raw material, the extraction conditions and also the storage of final product [35,36].

DPPH is the simplest and most widely used method for determining the free radical scavenging capacity. As expected, the high free radical scavenging activity of the extracts can be explained by the presence of phenolic compounds in extract composition, and the possibility of a synergistic effect between them, contributing to a stronger activity. This is of a great importance for the industry, as it may lead to more applications of *S. nigra* flowers as bioactive ingredient in food and pharmaceutical formulations, as an example. This claim is supported by other researchers who previously demonstrated that aqueous extracts of the *S. nigra* flower have a high antioxidant activity by different mechanisms of action evaluated from various methods (DPPH, FRAP, ABTS, etc.) [13,34,37]. For example, in a study by Viapiana et al. [34] the infusions of *S. nigra* L. flower extracts showed a radical scavenging activity by the DPPH method between 0.57–0.92 mmol TE/g of dry flower. These authors show greater activity for *S. nigra* flower extracts compared to our extracts, possibly due to differences in the extraction of phenolic compounds.

In a review, Ferreira et al. [13] reported variations in antioxidant activity of some elderflower extracts, and justifies these differences by plant growth conditions, post-harvest storage, extraction techniques, antioxidant evaluation test, etc., which may also explain the differences among our results.

### 4.2. Chemical Composition of S. nigra Extract

The extraction process was followed by identification and characterization of *S. nigra* extract using two chromatographic analytical techniques (GC-MS and HPLC-MS).

The study of metabolite profiling of the lipophilic fraction of *S. nigra* extract was achieved by GC-MS as shown in Table 1. This fraction presented a low recovery yield from the aqueous extract (0.42%) due to the extraction method, particularly to the use of water as initial solvent. A solute will dissolve in a solvent of identical polar property, but two substances of opposing polar properties will not interact, which means that nonpolar molecules, such as fatty acids, will not dissolve in polar substance like water [38]. Despite the low yield, these results provide insights on the *S. nigra* flowers’ composition, and may be used as an indication of further potential to deliver other molecules with different functionalities, provided that an appropriate extraction method and solvent are applied.

The major compound detected on the GC-MS was naringenin, a flavanone, which has received considerable attention regarding its pharmacodynamic activities [39,40]. These include: antioxidant activity, through the increase of antioxidant enzymes [41]; anti-inflammatory activity [42]; anticancer activity, namely by the suppressive effect on TGF-β signaling pathway, and so cytotoxic effect in many cell lines, such as colon, pancreas, leukemia, stomach, liver and breast [43,44]; anti-diabetic activity [45]; and effects on central nervous system and on cardiovascular system [46,47,48,49,50]. According to the literature, naringenin is a flavonoid compound present in elderflower extracts. As an example, Mikulic–Petkovsek and collaborators demonstrated the presence of this flavanone in methanolic extracts at higher concentrations than those obtained in our aqueous extract (734 µg g^−1^ vs. 122 µg g^−1^ of elderflower, respectively) [51]. This can be due to the chemical characteristics of naringenin, as this molecule is freely soluble in organic solvents like ethanol, methanol, dimethylformamide and dimethyl sulfoxide; however, in aqueous buffers naringenin is sparingly soluble, as performed in this study.

Other compounds present in the extract like phenolic acids, sugars and fatty acids increase the bioactive potential of the extract, particularly due to a possible synergistic effect among the compounds.

MCFAs and LCFAs were detected on the GC-MS analysis. LCFAs are molecules with a carboxylic group (-COOH) with a long aliphatic chain. The carboxylic group has the capability to form strong hydrogen bonds, namely the low members of the family (C_1_–C_6_), however, as the hydrocarbon chain increases there is a loss of solubility [38]. So, it would be expected to find higher concentration of short-chain fatty acid (SCFAs), instead of MCFAs and LCFAs. However, no SCFAs were detected in GC-MS analysis. We suggest that during dichloromethane fractioning, SCFAs were maintained in the water layer and were not transferred to the dichloromethane layer, due to their high solubility in water.

Several studies reported the lipids content and fatty acids profile from *S. nigra* berries [52], although to our knowledge the use of lipids from *S. nigra* flowers is quite limited. Taken all together, our results suggest that *S. nigra* flowers are a value source for the intake of beneficial fatty acids in humans’ diets if used as nutrient sources in food products. Furthermore, some of the identified fatty acids can be used for medical applications, such as in the treatment of dermatitis, diabetes, and inflammatory diseases [53].

The phenolic profile of *S. nigra* aqueous extract (Table 2) assessed by HPLC-MS is consistent with literature, in particular the highest content of chlorogenic acids (quinic acid; caffeoylquinic acid; dicaffeoylquinic acid; coumaroylquinic acid; feruloylquinic acid), rutin, quercetin, isorhamnetin and kaempferol. Flavonols derived from *S. nigra* flowers mainly occur as glycosides of rutin and glucose; moreover, acylated quercetins were also present. For example, the concentrations of flavonoids and their glycosides determined in the inflorescence of *S. nigra* present by Barros et al. [54] are in agreement with our results. However, the content of phenolic acids (especially isomers of chlorogenic acid) are higher, while the amount of flavonoid rutin is lower [54,55]. The composition of *S. nigra* aqueous extract demonstrates that it might be a source of valuable bioactive compounds, and particularly of phenolic acids and flavonols. As previously reported in the results section, this extract has a high concentration of caffeoylquinic acid, quercetin, isorhamnetin and kaempferol. The caffeoylquinic acid and its derivatives have shown promising biological activities such as antioxidant, immunomodulatory, antihypertensive, analgesic, anti-inflammatory, hepato- and neuroprotective, anti-hyperglycemic, anti-cancer, antiviral and antimicrobial [56,57]. Quercetin and kaempferol also have similar properties, highlighting their antioxidant, anti-inflammatory, anti-cancer and antibacterial activity [58,59,60,61]. Isorhamnetin derivatives have reported important antioxidant and anti-cancer actions [62,63]. As mentioned earlier, the biocompounds present in *S. nigra* extract can have a synergistic effect, influencing their action and application as a nutraceutical or food additive.

### 4.3. In-Vitro Cell Assays and Antimicrobial Activity

Colorectal cancer remains a major cause of cancer related death and diet is known to be a determinant factor in the disease’s etiology. Diets rich in fruits and vegetables are associated with lower rates of colorectal cancer, mainly due to their richness in fiber, although antioxidants (e.g., polyphenolic compounds) may also exert some influence. DNA protection from oxidative damage can contribute to cancer prevention or delayed progression maintain the genomic stability. Therefore, modulation of proliferative behavior, as well as, DNA protection and/or induction of repair are important parameters for anticancer therapy.

*S. nigra* extract was tested in three different cell lines. The antiproliferative effect of *S. nigra* aqueous extract differs according to the cell line tested, being RKO the most susceptible line and HCT116 the least susceptible (see Table 3), but overall, *S. nigra* aqueous extract exhibited low antiproliferative activity, when compared to other plant extracts [64]. These results are in accordance with some studies regarding to the anti-inflammatory and anticancer properties of *S. nigra* using cyclooxygenase (COX)-1, COX-2, quinone reductase, and ornithine decarboxylase assays at 10 µg mL^−1^ of extract [65]. However, some studies reported that extracts from *S. nigra* leaves and fruits moderately inhibited tumor growth in colorectal cancer cells (HT29) and leukemia, being these antitumor properties associated with the polyphenol content [66,67,68].

In a recent study by our group IC_50_ of vine pruning hydroethanolic extract was found to be 21 µg mL^−1^ in RKO cells which is considerably lower for the same cell line and IC_50_ for HCT116 (53 µg mL^−1^) were also considerably lower than those for *S. nigra* aqueous extract [69]. Water extracts often produce lower anti-proliferative effects than ethanolic extracts, for example, due to their hydrophilic nature and limited membrane crossing ability.

As mentioned before, several compounds detected in *S. nigra* extract have implication in cancer therapy as chemopreventive agents or as supplements that act as adjuvants in chemotherapy, namely quercetin, caffeoylquinic acid, naringenin and kaempferol. These compounds and their derivatives are known to exert growth inhibitory effects in colon cancer cells by decreasing tumor growth and suppressing cell survival or proliferation rate through induction of apoptosis or autophagy [70,71,72,73]. Additional according to the literature other natural compounds present in plant-based products, such as resveratrol, calebin A or curcumin are activated as intracellular signaling molecules during anti-inflammatory and anti-tumor action, via multiple signaling pathways [74,75,76]. These compounds can act synergistically with other compounds present in the diet that can enhance the effect and improve the therapeutic action of the biomolecules.

On the other hand, knowing the chemical complexity of *S. nigra* flower extract, we can assume that this set of compounds has a broad effect, showing different implications and their role as a multi-target source in the prevention and treatment of cancer.

Due to the chemical composition (Table 1 and Table 2) and antioxidant activity of the extract produced at 90 °C (Figure 1B), its ability to prevent oxidative DNA damage or to induce oxidative DNA repair was tested in RKO cells using the comet assay. This assay detects DNA strand breaks induced by an oxidizing agent, like H_2_O_2_. Fragmented DNA migrates in an electrical field creating a cloud similar to the tail of a comet [21]. The percentage of DNA in the tail is directly proportional to the fragments present and the higher the number of fragments the larger the tail. In two separate versions of the assay two different effects of the extract may be addressed: (i) the capacity to prevent DNA damage in response to H_2_O_2_ exposure or (ii) the effects of the extract on the induction of the cells’ DNA repair capacity. In the first case, damage induced by H_2_O_2_, cells previously incubated with the extract should present fewer fragments and lower DNA in comet tail in comparison to non-incubated cells with the extract, if the extract had protective effects on DNA oxidative damage. An effect that was not observed in this study.

Numerous reports have shown that polyphenols, such as quercetin, a molecule present in elderflower extract, although not a major constituent, increases DNA repair activity at a concentration above 20 µM. Although, it has a cytotoxic effect at 100 µM in HepG2 cells [77]. Caffeoylquinic acid and derivatives, the most abundant compounds present in the tested extract, have been shown to possess anti-colon cancer effects in HT-29 cells promoting cell death by apoptosis at concentrations of 5 and 10 µM [78]. However, in the present study, we observed that *S. nigra* extract at non-cytotoxic concentrations (200 and 400 µg mL^−1^) did not protect RKO cells from the oxidative activity of H_2_O_2_ or induce DNA repair activity.

In the comet assay, to assess the effect of the extract repair ability, after exposure to H_2_O_2_, the cells are allowed to recover to repair their DNA. Calculating the repair capacity, the higher the bar in the graph in Figure 4, the faster the repair is occurring. The data presented in Figure 5 shows that pre-treatment with luteolin (the positive control) results in significant induction of DNA repair, but the extract did not demonstrate to have the ability to induce DNA repair. Luteolin is a phenolic compound with known anticancer activity. Luteolin’s anticancer property is associated with the inhibition of cell proliferation, metastasis, angiogenesis, and induction of apoptosis. Luteolin sensitizes cancer cells to therapeutic-induced cytotoxicity, through suppressing cell survival pathways like, phosphatidylinositol 3′-kinase (PI3K)/Akt, nuclear factor kappa B (NF-κB), X-linked inhibitor of apoptosis protein (XIAP), and stimulating apoptosis pathways (the tumor suppressor p53) [79,80].

The small effects on DNA damage prevention and repair observed, in spite of the demonstrated antioxidant potential of the aqueous extract, may reflect the hydrophilic nature of the compounds present. This hydrophilic nature decreases the ability of the molecule to penetrate the cell’s membranes and therefore not reach the DNA in the nucleus [81].

Since the earliest times, many plants have been known to exert healing properties against human pathogens, due to their secondary metabolite content. Over the past decade, much attention has been placed on the study of phytochemicals for their antibacterial/antifungal activity [82]. Although, phytochemicals cannot be used in monotherapy, due to their high minimum inhibitory concentration (MIC) (100–5000 µg mL^−1^) compared with antibiotics (0.031–512 µg mL^−1^) [83], they are also therapeutically relevant because they can modulate or modify resistance mechanisms in bacteria. Therefore, phytochemicals can be used in combination with antibiotics to increase the activity and decrease the doses of antibiotic [84]. Based on our results, *S. nigra* extract has therapeutic potential as antimicrobial agent (MIC of 4100 and 8300 µg mL^−1^) and this potential may arise from their components.

Besides, flavonols antioxidant, anti-inflammatory, antiallergic, anticancer and antimicrobial properties, they exhibited antimicrobial activity. This activity is due to the ability of plants to synthetize molecules with anti-microbial activity to response a microbial infection [27,85]. Among flavonoids, flavonols, such as quercetin and kaempferol have shown antimicrobial activity against Gram-negative and Gram-positive bacteria [86,87].

Phenolic acids have recently gained substantial attention due to their various functional, biological and pharmacological effects. For instance, several studies indicate that chlorogenic acid has an effect on *K. pneumoniae* [83,88], *P. aeruginosa* [83], *S. epidermis* [89] and *S. aureus* [90]. Also kaempferol was previously described as effective against *S. aureus* (at a concentration of 22 μM) [91]. Despite the abundance of flavonoids and other phenolic compounds found in *S. nigra* aqueous extract (Table 2), in our study *S. nigra* extracts seemed to be particularly effective against Gram-positive bacteria, namely against staphylococcal species, in opposite to Gram-negative. This distinct activity of phenolic extracts between Gram-positive and Gram-negative was also previously reported in the literature [2,92,93]. The mechanism of action of several compounds of plant extracts, like naringenin, quercetin and kaempferol might not be due to selectively bacteria targeting, due to membrane disruption [94]. Consequently, the increased tolerance of Gram-negative bacteria to the action of plant extracts maybe related to cell membrane physiology. Gram-negative bacteria has a two-layer cell membrane with increasing hydrophilicity of the outer membrane. This effect is due to the presence of lipopolysaccharide molecules, providing a barrier to the penetration of molecules [93]. Small hydrophilic solutes can indeed overcome the outer membrane entering the cell through porin proteins, but macromolecules and most of the hydrophobic compounds are not able to penetrate the membrane.

It is of particular importance that phenolics, such as chlorogenic acid, are not effective against probiotic bacteria, making them even more appropriate for the food industry [95].

Chlorogenic acid can also exert antifungal effect against *Candida albicans* by having an impact on fungi’s cell membrane [96]. Interestingly, a lower MFC of *Candida albicans* would be expected, since it was described previously a MFC of 80 µg mL^−1^ [96], but in our extract the concentration reached 33,000 µg mL^−1^, which represents a of chlorogenic acid concentration of 246 µg mL^−1^.

Overall, results showed that *S. nigra* flower extract is a promising antimicrobial drug against the gram-positive bacteria, namely, against *S. aureus* and *S. epidermidis* (Table 4).

The antimicrobial action of *S. nigra* extract against staphylococcal species, may be due to the synergy of several compounds, including quercetin, kaempferol and chlorogenic acid. Nevertheless, the compounds responsible for this efficacy and the underlying mechanism of action will be addressed in a near future.

**In conclusion**, *S. nigra* flowers have a great potential as a good source of low-cost natural antioxidants with low cytotoxicity. The resulting extracts are very rich in polyphenols, particularly rutin, quercetin, caffeoylquinic acid and kaempferol, which are, at least partially, responsible for the exerted antioxidant, antiproliferative and antibacterial activities. These results demonstrated the potential of *S. nigra* flower extracts as a food additive, a cosmetic and for therapeutic applications. However, further in-vitro and in-vivo studies will need to be conducted to confirm the mechanisms of action and the health-promoting effects of *S. nigra* extracts.

## Figures and Tables

**Table 1:** Composition of *S. nigra* flower dichloromethane extract with the respective relative standard deviation (RSD).

Rt ^(A)^	(*m*/*z*)	Compound	µg g^−1^ Flower ^(B)^	µg g^−1^ Extract ^(B)^	RSD (%)
5.58	90.12	2–3-Butanediol ^(E)^	1.98	6.31	15.25
14.21	73, 117, 147, 186, 205	Glycerol	3.02	9.62	10.61
51.28	73, 129, 147, 203, 267	Glycerol monostearate	20.07	63.92	13.45
***Fatty acid***					
6.12	45, 75, 117, 173	Hexanoic Acid ^(C)^	3.12	9.94	12.57
16.27	215, 117	Nonanoic Acid ^(C)^	4.86	15.48	34.67
35.8	73, 117, 145, 313	Palmitic Acid ^(C)^	18.55	59.08	12.32
39.62	339, 117, 129, 145	Oleic Acid ^(C)^	5.95	18.95	11.51
40.32	73, 117, 145, 201	Stearic Acid ^(C)^	12.01	38.25	15.78
48.33	73, 117, 145, 201	Behenic Acid ^(C)^	5.53	17.61	3.64
52.04	73, 117, 145, 201	Lignoceric Acid ^(C)^	0.52	1.66	21.44
***Aminoacid derivatives***					
10.12	45, 73, 100, 160	Methyl leucinate ^(E)^	12.65	40.29	9.66
***Phenolic Acids and Tyrosol***					
11.77	51, 77, 105, 135, 179	Benzoic Acid ^(D)^	8.83	28.12	8.22
20.73	64, 77, 107, 135, 209	ρ-Anisic Acid ^(D)^	23.91	76.15	8.96
21.52	205, 131, 161	Cinnamic acid ^(D)^	4.97	15.83	8.25
32.88	73, 117, 147, 179, 219, 249, 293, 308	4-Coumaric Acid ^(D)^	8.17	26.02	5.28
36.53	73, 117, 147, 191, 219, 249, 293, 323, 338	Ferulic Acid ^(D)^	22.79	72.58	8.10
23.04	73, 103, 147, 267	Tyrosol ^(E)^	4.30	13.69	7.26
***Flavonoid***					
50.81	73, 117, 151, 179, 224	Naringenin ^(E)^	30.37	96.72	10.63
52.28	73, 133, 147, 179, 229, 268	Naringenin ^(E)^	91.82	292.42	12.19
***Monoterpene***					
21.74	73, 131, 157, 199, 227	Linolool Oxide ^(E)^	15.83	50.41	8.52
26.45	73, 131, 143, 183	Linolool Oxide ^(E)^	12.34	39.30	8.67
***Sugar***					
20.01	73, 133, 147, 189, 204	Rhamnose ^(E)^	10.09	32.13	8.57
46.93	73, 129, 147, 189, 204	Cellobiose ^(E)^	31.63	100.73	7.36

^(A)^ Rt.—retention time (min); ^(B)^ Content values are expressed as mean ± RSD, *n* = 3; Calibration was performed using ^(C)^ palmitic acid, ^(D)^ ferulic acid, or ^(E)^ tetracosane.

**Table 2:** Phenolic compounds identification in *S. nigra* flower aqueous extract by HPLC and corresponding MS^n^ fragmentation profiles.

Peak	Rt ^(A)^	λ_máx_ (nm) ^(B)^	Compound	[M–H]^−^ (*m*/*z*) ^(C)^	MS^2^ (*m*/*z*) ^(C)^	MS^3^ (*m*/*z*) ^(C)^	µg g^−1^ of Flower ^(E)^	µg g^−1^ of Extract ^(E)^
1	0.60	238, 255	Quinic Acid ^(a)^	191	[191] ^(D)^: 85 (37), 87 (22), 109 (23), 111 (100), 127 (57), 171 (29), 173 (41)	_	545.0 ± 27.7	1735.8 ± 88.1
2	0.71	249, 259	*p*-Coumaroyl-caffeoylquinic Acid ^(a)^	499	[499]: 191 (100), 173 (57), 481 (18)	[191]: 85 (91), 171 (100)	747.7 ± 105.7	2381.2 ± 336.6
3	4.34	246, 292, 322	Caffeoylquinic Acid ^(a)^	353	[353] ^(D)^: 179 (34), 191 (100), [179]: 135 (100)	_	879.3 ± 29.6	2800.3 ± 94.3
4	5.48	234, 288	Dicaffeoylquinic Acid ^(a)^	515	[515]: 191 (100), 352 (52), 379 (28)	[353]: 191 (100)	145.7 ± 41.8	464.0 ± 133.1
5	6.04	253, 308, 358	Caffeoylquinic Acid ^(a)^	353	[353]: 191 (100)	[191]: 85, 93 (54), 111 (40), 127 (72), 171 (49), 173 (100); [173]: 87 (23), 93 (100), 111 (100), 155 (59)	7394.10 ± 253.6	23548.1 ± 807.7
6	6.48	241, 293, 324	Caffeoylquinic Acid ^(a)^	353	[353]: 173 (100), 179 (49), 191 (20), [173]: 93 (100), 109 (36), 137 (76), 155 (73)	_	873.1 ± 79.4	2780.6 ± 252.9
7	7.08	233, 286, 321	Quercetin dihexoside ^(b)^	625	[625]: 301 (51), 462 (24), 463 (100)	[301]: 151 (100), 179 (73), 231 (41)	181.2 ± 38.3	577.1 ± 121.9
8	7.54	236, 288, 308	Coumaroylquinic Acid ^(a)^	337	[337]: 191 (100), 173 (22)	[191]: 127 (100), 173 (85); [173]: 129 (21), 137 (29), 155 (100)	435.6 ± 37.9	1387.3 ± 120.7
9	8.03	233, 264	Coumaroylquinic Acid derivative ^(a)^	401	[401]: 269 (100), 191 (37)	[269]: 113 (21), 159 (23), 161 (100)	93.6 ± 15.0	298.1 ± 47.8
10	8.38	237, 312, 321	Feruloylquinic Acid ^(a)^	367	[367]: 173 (45), 191 (100)	_	367.5 ± 2.7	1170.4 ± 8.59
11	8.62	233, 254, 334	Quercetin dihexoside ^(b)^	625	[625]: 255 (21), 271(24), 300 (100), 301 (61), 445 (53)	[301]: 151 (58), 179 (100)	293.1 ± 43.4	933.4 ± 138.2
12	9.05	234, 254, 334	Quercetin trisaccharide ^(b)^	755	[755]: 271 (25), 300 (100), 301 (33), 343 (39), 505 (31), 591 (79), 609 (45)	[301]: 151 (26), 179 (100); [609]: 283 (69), 300 (32), 343 (100)	327.8 ± 33.5	1043.9 ± 106.7
13	9.97	233, 254, 343	Isorhamnetin derivative ^(b)^	639	[639]: 299 (27), 315 (100), 459 (29)	[315]: 300 (100)	273.4 ± 31.5	870.7 ± 100.3
14	10.12	254, 318, 374	Quercetin-3-rutinoside ^(b)^	609	[609]: 301 (100), 300 (42)	[301]: 179 (100), 151 (69)	1887.7 ± 269.1	6011.8 ± 857.0
15	11.85	232, 255, 350	Quercetin-acetyl glucoside ^(b)^	505	[505]: 300 (62), 301 (100), 463 (23)	[301]: 151 (100), 179 73), 193 (24), 273 (46), 283 (23)	413.9 ± 30.4	1318.2 ± 96.8
16	12.73	232, 264, 343	Kaempferol rutinoside ^(b)^	593	[593]: 285 (100)	[285]: 197 (24), 213 (25), 229 (45), 241 (21), 257 (100), 267 (54)	286.1 ± 30.1	911.1 ± 95.9
17	13.68	234, 254, 343	Isorhamnetin-rutinoside ^(b)^	623	[623]: 300 (23), 315 (100)	[300]: 255 (67), 271 (100), 272 (25)	601.2 ± 27.7	1914.7 ± 88.2
18	14.64	233, 301, 385	Kaempferol residue ^(b)^	449	285 (100), 303 (23)	[285]: 141 (100), 123 (23)	288.8 ± 23.7	919.7 ± 75.5
19	15.94	232, 254, 348	Isorhamnetin acetylhexoside ^(b)^	519	271 (30), 314 (100), 315 (72), 357 (36)	[315]: 300 (100)	281.4 ± 27.7	896.2 ± 88.2

^(A)^ Rt.—retention time (min); ^(B)^ λ_máx_—maximum wavelength; ^(C)^ MS^2^, MS^3^—second and third stage of mass spectrometry; ^(D)^ […]—product ions were subjected to further MS^3^ fragmentation; ^(E)^ Content values expressed as mean ± SD, *n* = 3. Calibration curves used: ^(a)^ chlorogenic acid and ^(b)^ rutin.

**Table 3:** IC_50_ values for *S. nigra* flower aqueous extract on Human colorectal cancer cell lines.

Cell Lines		
Caco-2	RKO	HCT-116
2520 ± 310	1250 ± 60	3441 ± 230

IC_50_ values are presented in µg mL^−1^. Results are expressed as mean ± SD of at least three independent experiments.

**Table 4:** MBC and MFC performance of *S. nigra* flower aqueous extract against pathogenic organisms.

Microbial Species	Strain	MBC (µg mL^−1^)
**Bacteria**		
*Pseudomonas aeruginosa*	PA01	>33,000
*Klebsiella pneumonia*	ATCC 11296	>33,000
*Klebsiella oxytoca*	ATCC 13182	>33,000
*Staphylococcus aureus*	ATCC 25923	8300
*Staphylococcus epidermidis*	ATCC 12228	4100
**Fungi**	**Strain**	**MFC** **(µg mL^−1^)**
*Candida albicans*	SC 5314	>33,000

MBC and MFC values are presented in µg mL^−1^. Results are expressed as mean of at least three independent experiments.

## Data Availability

Not applicable.

## Supplementary Materials

The following are available online at https://www.mdpi.com/article/10.3390/biom11081222/s1, Figure S1: Cellular viability (%) estimated by MTT assay of *S. nigra* flower aqueous extract against human colon carcinoma (RKO), human colorectal carcinoma (HCT116), human colorectal carcinoma (Caco-2) cells. Data are expressed as mean ± S.D. (*n* = 3).
