# Supplementary material for: Chemical Characterization of Sambucus nigra L. Flowers Aqueous Extract and Its Biological Implications
Source: Biomolecules. 2021 Aug 17;11(8):1222. doi: 10.3390/biom11081222 (PMC8391949; doi:10.3390/biom11081222)
Supplement: Supplementary file 1 [file biomolecules-11-01222-s001.zip › biomolecules-1276731-supplementary.pdf]

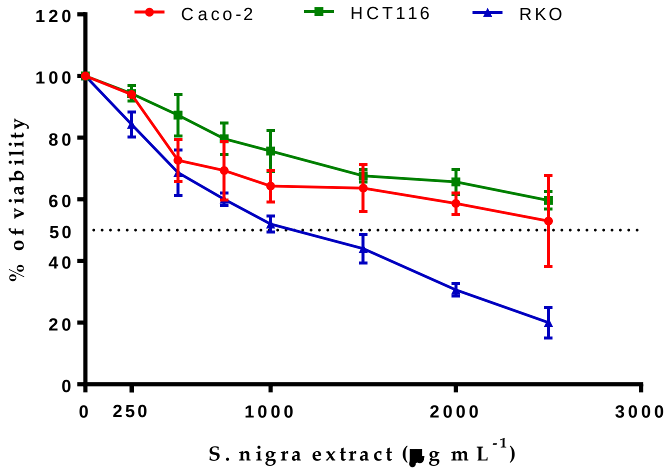

Figure S1. Cellular viability (%) estimated by MTT assay of *S. nigra* flower aqueous extract against human colon carcinoma (RKO), human colorectal carcinoma (HCT116), human colorectal carcinoma (Caco-2) cells. Data are expressed as mean ± S.D. (n=3)
